# The Families Improving Health Together (FIT) Program: Initial evaluation of retention and research in a multispecialty clinic for children with obesity

**DOI:** 10.1002/osp4.498

**Published:** 2021-03-23

**Authors:** Michael Rosenbaum, Robert Garofano, Kalle Liimatta, Kerry McArthur, Erin Paul, Thomas Starc, Aviva B. Sopher, Vidhu Thaker, Jennifer Woo Baidal

**Affiliations:** ^1^ Berrie Diabetes Research Pavilion Columbia University Medical College New York USA

**Keywords:** child, interdisciplinary research, multispecialty treatment, obesity

## Abstract

**Background:**

Obesity affects ∼17% of US children, with parallel increases in multiple comorbidities, especially among African‐, Asian‐, Hispanic‐, and Native‐Americans. Barriers to patient retention in pediatric obesity programs include lack of centralized care, and frequent subspecialty MD visits which conflict with patient school attendance and parental work attendance as well as with support service utilization. Lack of integration of multispecialty clinical care with interdisciplinary research is a major barrier to fuller exploration of the treatment, prevention, and understanding of obesity in childhood.

**Objective:**

To test the hypothesis, a novel multispecialty/interdisciplinary clinical and research infrastructure with strong emphasis on a primary obesity care physician for children with early‐onset (<9 years) obesity (Families Improving health Together [FIT]) could promote lower patient attrition (primary goal) and foster productive research in pediatric obesity (secondary goal).

**Results:**

Data support the hypotheses. Over 15 months, FIT reported a >90% participant retention (*p* < 0.001 vs. expected rate based on other studies of similar programs). Though 90% of children had at least one adiposity‐related comorbidity and 70% had at least two, there was no need for additional subspecialist visits with cardiologists, endocrinologists, gastroenterologists, or molecular geneticists. Three abstracts were presented at national meetings, and two manuscripts were published all with junior faculty as primary authors.

**Conclusion:**

This pilot study suggests that an integrated multispecialty/interdisciplinary approach to children with obesity improves patient retention and can be integrated successfully with research.

## 1 INTRODUCTION

Care for obesity and its comorbidities currently accounts for over $200 billion per year in healthcare costs (21% of total US healthcare budget) in the United States.^1^ In New York State (NYS), 16%–17% of toddlers and adolescents have overweight and 11% of adolescents and 15% of toddlers have obesity.^2^ The likelihood of sustained medically significant nonsurgical weight loss in adults has remained about 12%–20%,^3^, ^4^, ^5^ despite multiple new interventions. The duration of reduced 10% or greater body fatness (by body mass index [BMI] *z*‐score) is significantly improved in prepubertal children (success rates up to ∼50%) compared to adults enrolled in structured weight loss programs.^6^, ^7^, ^8^, ^9^, ^10^

Obesity represents a complex interaction of biological, social, and environmental problems that require integrated care and research beyond individual discipline boundaries or “silos.”^11^, ^12^ These silos may limit the coordination of clinical care and the collaboration of researchers to evaluate obesity as a multisystem disease.^12^, ^13^ The National Institutes of Health (NIH) has recognized the need for combined clinical care and interdisciplinary translational research ranging from individual microsystems, including genetics, to environmental macrosystems.^14^, ^15^, ^16^, ^17^, ^18^

There are a number of suggested approaches to better integrate multispecialty clinical care and interdisciplinary research for children with obesity by diminishing barriers to participant retention. From a patient standpoint, compartmentalized referrals for each comorbidity (frequent MD visits), even within the same program, have been associated with poor child and family retention in treatment clinics.^19^, ^20^, ^21^, ^22^, ^23^ Subspecialty referrals increase time demands and costs (missed school days and work days) for affected children and their families and diminish time available for participation in support services (e.g., dietary and exercise programming). In addition, noncentralized care often results in poor communication among physicians managing obesity, specialists managing comorbidities, dietitians, exercise programs, and social workers. From a provider standpoint, the most frequently reported barriers have included lack of time (75%), lack of awareness of referral options (70%), lack of coordinated care (58%), and costs (71%–85%).^24^ These data suggest rates of attrition might be reduced in a program based on a single primary care physician who coordinates all care to minimize referrals and maximize communication regarding the child with obesity.^25^, ^26^, ^27^, ^28^

Families Improving Health Together (FIT) was a new program that was operational at the Children's Hospital of New York at Columbia University Irving Medical Center from March 2017 to July 2018. FIT focused on centralizing care of patients with obesity to a single physician supported by an on‐site clinical multispecialty and academically interdisciplinary team. The interdisciplinary approach was distinct from other institutional subspecialty‐based programs in which comorbidities frequently required referral to other subspecialists. This is a pilot study of a novel means to increase retention and encourage multispecialty/interdisciplinary research in a program for children with obesity. The primary hypothesis was that centralization of care for pediatric obesity would reduce the need for referrals, diminish attrition, and increase interactions with the primary care obesity physician, dietitian, and exercise programming. A secondary hypothesis was that an integrated research and clinical environment with junior and senior faculties as well as standardized enrollment forms, a mineable database, and presentation of research goals would breakdown academic silos and stimulate interdisciplinary research.

## 2 METHODS

### 2.1 Funding

The FIT program was implemented at the Children's Hospital of New York at Columbia University Irving Medical Center with funding from the NY State Empire Clinical Research Investigator Program. FIT was funded as a pilot study to determine whether a multispecialty, interdisciplinary approach would improve patient retention and research. These results would then be incorporated into the infrastructure of a larger, more sustained, pediatric obesity initiative.

### 2.2 Initial FIT design

Prior to opening the FIT clinic, the following resources were developed:

1. An overview curriculum discussing clinical research design, ethics, and statistical analyses for young investigators.
2. An extensive screening questionnaire (see Supplement) for each age group (ages 2–4, 5–7, and 8–10 years).
3. A database that interacted with the electronic medical records to allow direct downloading of vital signs, laboratory work, and ongoing treatment recommendations, as well as the data from the screening questionnaire.
4. Research projects for each junior faculty/senior faculty member pair. Specifics were precursors of metabolic syndrome in childhood obesity; vitamin D deficiency prevalence and treatment in children with obesity; risk factors for fatty liver disease in children with obesity, and gene dose effects of FTO single nucleotide polymorphisms (SNPs) on feeding behavior and neuronal connectivity; and an outline of community outreach planning.
5. Seminar schedule to familiarize all healthcare providers with issues related to obesity within their specialties and progress reports regarding their research.

### 2.3 FIT infrastructure

The FIT infrastructure consisted of a coordinator, physicians (senior and junior faculties and fellows), a registered dietitian, and an exercise physiologist. The coordinator was responsible for scheduling of patients, ascertaining that the prescreening questionnaire was completed before patients were seen (see below), and for arranging regular seminars for FIT physicians. The clinical team consisted of senior and junior faculty from the Divisions of Pediatric “Cardiology,” “Endocrinology,” “Gastroenterology, Hepatology, and Nutrition,” and “Molecular Genetics,” as well as a dietitian and an exercise physiologist. Primary FIT physicians were assistant professors, associate professors, or fellows, and were responsible for organizing patient care and presenting patients to the entire team as new data became available. This organizational format allowed the more senior advisory group to directly supervise fellows and, in general, to provide early career introductions to obesity care and research. The FIT program was conducted weekly in the Phyllis and Ivan Seidenberg Center for Nutritional Wellness at the New York Presbyterian Morgan Stanley Children's Hospital which included a bariatric scale and examination and waiting areas specifically constructed to accommodate individuals with obesity.

At the start of each clinical session, there was either a patient review of all patients who had been seen 2 weeks or more previously (to allow time for laboratory data acquisition) by the entire team including junior and senior faculties from each division and the dietitian (K.M.) or a seminar given by one of the faculties or by an outside investigator on some aspect of obesity in childhood.

### 2.4 FIT screening

Children with early‐onset obesity (BMI > 95%ile by age 9; ages 2–10 at enrollment) were referred by their primary care physicians. Primary care physicians were informed about FIT by advertisements that publicized the FIT website. Prior to arrival in the FIT clinic, parents were contacted by the FIT coordinator (K.L.) and asked to complete an extensive questionnaire (available in English and Spanish) to assess medical and family history, dietary habits, exercise habits, sleep habits, and the environment in which the children live (see Supplement). Growth charts, laboratory records, and any previous consultation records were obtained from the referring pediatrician before any appointment was made for a new patient in the FIT program. Written informed consent and assent forms were offered in English or Spanish to participate in FIT with options for biobanking of blood and DNA samples from participants and to be contacted regarding other research opportunities. All protocols, consents, and assents were approved by the Human Research Protection Office/Institutional Review Board.

### 2.5 FIT initiation

**FIGURE 1:**
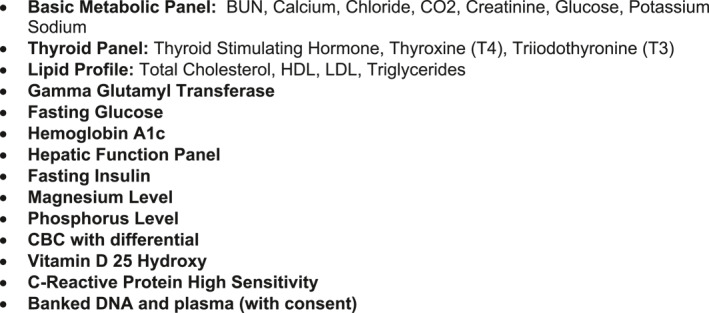
Initial laboratory screening. Routine fasting laboratory testing was done every 6–12 months unless the severity of a detected abnormal result (e.g., elevated glycosylated hemoglobin or thyroid disease) indicates the need for more frequent sampling

Each patient was assigned a primary FIT pediatrician from the division of pediatric cardiology (E.P.), endocrinology (A.S.), gastroenterology (J.W.B.), or molecular genetics (V.T.). This physician would remain as their primary contact and communicate all dietary and other recommendations from the entire team to each family. New patients met with their primary FIT physician for a complete physical and validation of questionnaire data and then with the FIT dietitian (total time about 90 min). Vital signs including waist circumference, body fat percentage by Bio‐electrical Impedance Analysis (BIA), and blood pressure (repeated three times) were measured for every child. Within 2 weeks of the initial visit, children were scheduled to return for fasting laboratory testing^29^ (see Figure 1).

### 2.6 FIT follow‐up

Parents returned to discuss the results of the screening laboratory tests with their FIT physician 2 weeks after labs were drawn. Prior to this, the entire FIT team, including the cardiology, endocrinology, gastroenterology, molecular genetics, and nutrition subspecialists, met to discuss the patient and their lab results. Team recommendations were communicated to parents by the FIT physician. Follow‐up appointments were then made to meet with the dietitian every 1–2 months and their FIT physician at 3–4 month intervals. Routine fasting laboratory testing was done every 6–12 months unless the severity of a detected abnormal result (e.g., elevated glycosylated hemoglobin or thyroid disease) indicated the need for more frequent sampling. This routine laboratory testing functioned as a measure of co‐morbidity presence/risk and as biomarkers that could be incorporated into discussions with families regarding the patient's health status. Letters were sent to referring primary care physicians after all MD visits.

### 2.7 FIT diet

Participants usually met for a full session with the registered dietitian (KM) at their first clinic visit. At this time, a dietary history (both family and participant) was taken and a relation was established wherein the dietitian would make culturally sensitive suggestions for a more healthful family diet and specifically to reduce caloric intake in the affected participant. Following the meeting with the FIT physician to discuss laboratory results, a visit with the dietitian was usually scheduled within 4 weeks depending upon the need for dietary adjustments based upon the laboratory results and participant feedback regarding initial recommendations. It was recommended that follow‐up visits with the dietitian occur every 1–2 months.

### 2.8 FIT exercise (HOP‐UP)

**FIGURE 2:**
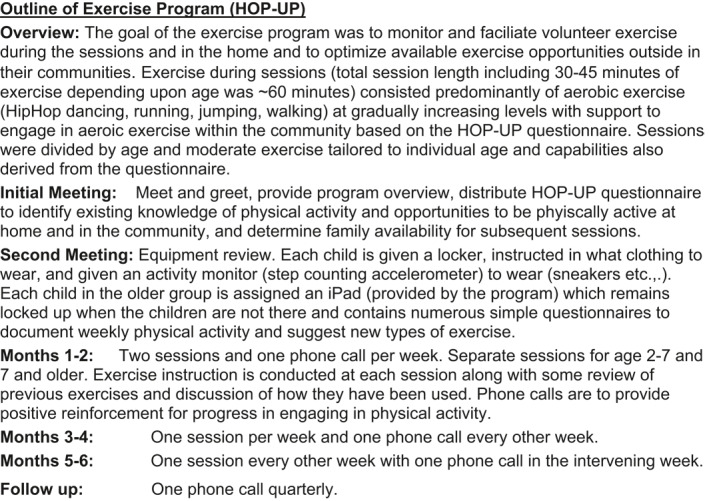
Outline of exercise intervention. The goal of this intervention was to give participants the knowledge to engage regularly in physical activity and acquaint them with the opportunities to do so in their communities

The FIT exercise program was named “FIT HOP‐UP” by Dr. Garofano who directed it. The goal of the exercise program was to monitor and facilitate participants' exercise during the sessions and in the home and to optimize available exercise opportunities in their communities. Exercise during sessions (total session length, including 30–45 min of exercise depending upon age, was ∼60 min) consisted of age‐appropriate moderate to vigorous physical activity (MVPA) at levels similar to what those recommended by the U.S. Department of Health and Human Services^30^ and others.^31^, ^32^ Sessions were divided by age and moderate exercise was tailored to individual age and capabilities based on data from the FIT questionnaires (see Appendix). The previously established HOP‐UP^33^ questionnaire was also used to identify and encourage utilization of available community exercise opportunities. Exercise compliance was monitored by telephone and by questionnaire (available in English or in Spanish). The exercise program outline is schematized in Figure 2.

### 2.9 FIT database

The FIT program data system was created by the data management team in the Statistical Analysis Center (SAC) in the Department of Biostatistics at Columbia University Medical Center's (CUMC's) Mailman School of Public Health (Mr. Richard Buchsbaum). The system employs the SAC's secure web‐based platform, which uses a Microsoft SQL Server database, Microsoft ASP. Net web scripting, JavaScript, and a variety of other tools. The system tracks referrals to the FIT program, related clinic and dietitian appointments, family history and cardiometabolic risk factors, and other relevant clinical data. The system incorporates utilities for importing and integrating data from multiple CUMC data systems, including appointment scheduling software and the Clinical Data Warehouse. It includes utilities to aid clinical care, such as generation of detailed pedigrees and notification of significant risk factors, and utilities to assist in research, including tracking of informed consent and modules for recruiting and collecting data on family members. This database resides on a secure and HIPAA compliant SAC system for the collection of both patient care and research data. Data are stored in password protected files on a P‐drive in a manner only available to investigators directly involved in this study.

### 2.10 Outcome variables and statistical analyses

The primary outcome variable was patient retention rates over 6 months (defined as at least one follow‐up visit with the dietitian and/or physician after the initial visit with both the physician and dietitian and return for fasting blood testing with discussion of results). Because of the limited time that the program was operative (total of 12 months), longer follow‐up data were not available for most participants. Secondary outcome variables were the number of successful research collaborations involving multiple investigators and multiple disciplines (defined as publications, grants, or abstract presentations at national or international meetings) and the number of patients who required referrals to subspecialty clinics to see a clinician other than their primary care obesity physician. An exploratory research project was to establish a means to share relevant information with the Washington Heights/Inwood community which was by the number of presentations to the surrounding health care community regarding the management of children with obesity in a primary care setting.

### 2.11 Statistics and calculations

Rates of retention, defined as at least one visit by the child to the FIT physician and/or dietitian after the initial assessment and laboratory testing visits, and referral from FIT were compared with data obtained from results of other pediatric obesity programs^19^, ^34^, ^35^, ^36^, ^37^ using binomial tests of proportions.^38^ There are, to our knowledge, no data available regarding frequency of subspecialty referral within a pediatric weight management program. Studies of primary care physicians report that 7%–25% of children with obesity are referred to subspecialists (excluding dietitians and pediatric obesity programs) with most referrals to pediatric endocrinologists.^21^, ^36^

## 3 RESULTS:

### 3.1 Participants

**FIGURE 3:**
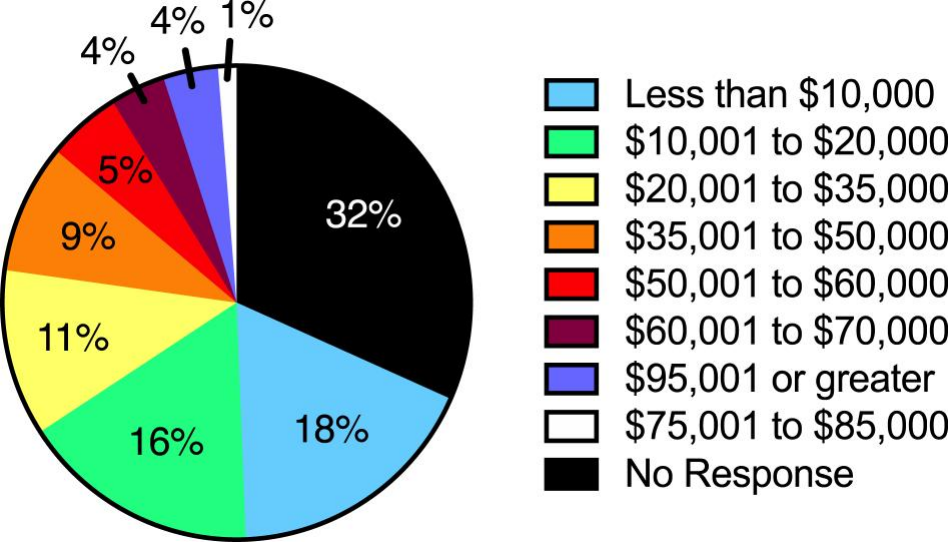
Annual household income (self‐reported)

The FIT program was funded by a 2‐year New York State Empire Clinical Research Investigator Award from 1 July 2016 to 30 June 2018. The initial 9 months was spent creating the program including a website, the database, and educational planning. Over the next 15 months, 106 participants inquired about FIT of whom 79 were seen with enough time to ascertain their retention rate (at least enough time for one scheduled patient follow‐up beyond the initial meeting with the physician and dietitian and a separate appointment for baseline [fasting] laboratory testing and discussion of results). The self‐reported ethnic/racial distribution of participants was 81% Hispanic (*n* = 64, 43% stated that Spanish was their preferred language), 10% Black (*n* = 8), 4% White (*n* = 3), 1% Arabian (*n* = 1), 1% multiracial (*n* = 1), and 3% of families were nonresponsive (*n* = 2). Participant characteristics are presented in Table 1. Socioeconomic data (annual household income) data were also requested and are schematized in Figure 3. Because of the low number of participants who self‐identified as non‐Hispanic and the high number who did not provide reliable data regarding family history of obesity and socioeconomic status (see Table 1 and Figure 3), these data were not analyzed as correlates of attrition or comorbidities.

**TABLE 1:** Mean (SD) and range of participant demographics

Demographics		All (*N* = 79)	Males (*n* = 41)	Females (*n* = 38)
Age (years) – Mean (SD)		6.6 (2.4)	6.6 (2.0)	6.9 (2.4)
Range		2.3–11.2	2.3–10.5	3.1–11.2
Age distribution (years)	2–4	22, 28%	13, 32%	9, 24%
(*N*, % of population)	5–7	36, 45%	18, 44%	18, 47%
	8–10	19, 24%	10, 24%	9, 24%
	11	2, 3%	0, 0%	2, 5%
BMI – Mean (SD)		27.5 (5.3)	27.1 (4.5)	26.9 (5.69)
Range		20.0–36.5	20.0–37.3	22.5–36.5
BMI % of the 95%ile – Mean (SD)Range		135.2 (19.8)	141.8 (22.1)	130.6 (16.3)
		105.9–193.2	105.9–193.2	108.5–176.0
One or more parents with obesity^*^ ^a^ (*n*, % of population)		44, 56%	21, 51%	23, 60%

*Self‐reported.

^a^ Data available on only one parent in 35% of participants.

### 3.2 Comorbidities

As shown in Table 2, over 90% of participants had at least one adiposity‐related co‐morbidity and over 70% had at least two comorbidities consisting of systolic and/or diastolic hypertension, dyslipidemia, evidence of insulin resistance, vitamin D insufficiency or deficiency, or laboratory evidence of nonalcoholic fatty liver disease (NAFLD). Even within this population of children with obesity, adiposity‐related co‐morbidity markers were often significantly correlated with adiposity (BMI *z*‐score, see Table 3).

**TABLE 2:** Mean (SD) values related to co‐morbidities and percentage classified as having adiposity‐related co‐morbidities

Adiposity‐related comorbidities		All (*N* = 79)	Males (*n* = 41)	Females (*n* = 38)	% abnormal	Definition
Systolic BP		104 (10)	105 (11)	104 (10)	43% (*n* = 34)	>90%ile for age and sex
Diastolic BP		65 (8)	66 (7)	64 (8)	39% (*n* = 31)	
Glucose (mg/dl)		85 (7)	86 (7)	84 (8)	0% (*n* = 0)	>100
Insulin (mIU/ml)		12.4 (8.9)	11.9 (9.6)	12.8 (8.4)	66% (*n* = 52)	>7.0
Hba1C		5.1 (0.3)	5.2 (0.3)	5.1 (0.3)	6% (*n* = 5)	>5.7
Cholesterol (mg/dl)	Total	156 (34)	165 (40)	**148 (27)** *	36% (*n* = 28)	>170
	Triglyceride	99 (54)	89 (50)	108 (56)	56% (*n* = 44)	>75
	HDL	47 (11)	50 (13)	44 (10)	19% (*n* = 15)	<35
	LDL	89 (27)	98 (31)	**81 (20)** *	25% (*n* = 20)	>100
Vitamin D (ng/ml)		24.9 (6.5)	24.3 (7.8)	25.3 (5.2)	83%^a^ (*n* = 66)	<30
AST (U/L)		29.3 (11.7)	30.2 (14.7)	28.5 (8.2)	17% (*n* = 13)	>90%ile for age and sex
ALT (U/L)		22.6 (14.3)	21.7 (14.2)	24.4 (14.4)	14% (*n* = 11)	
Alkaline phosphatase (U/L)		275 (68)	248 (48)	**301 (74)** *	30% (*n* = 24)	

*Note:* Statistically significant differences from males are highlighted **in bold.**

Abbreviations: ALT, alanine aminotransferase; AST, aspartate aminotransferase.

^*^ *p* < 0.05 versus males.

^a^ 55.6% of the participants were vitamin D insufficient (20 ng/ml < 25‐hydroxy vitamin *D* < 30 ng/ml) and 18.5% were vitamin D deficient (25‐hydroxyl vitamin *D* < 20 ng/ml).

**TABLE 3:** Correlations of adiposity‐related co‐morbidity assessments and adiposity measured by BMI % above the 95%ile

Adiposity and comorbidity correlations	*R*	*p*
Variable		
**BMI**	**0.62**	**<0.001**
**Systolic BP**	**0.36**	**0.002**
**Diastolic BP**	**0.34**	**0.005**
**Glucose (mg/dl)**	**0.27**	**0.036**
**Insulin (mIU/ml)**	**0.65**	**<0.001**
**Hba1C**	**0.47**	**<0.001**
Total cholesterol (mg/dl)	0.15	0.28
Triglycerides (mg/dl)	0.05	0.82
HDL cholesterol (mg/dl)	0.02	0.84
LDL cholesterol (mg/dl)	0.15	0.17
Vitamin D (ng/ml)	−0.23	0.06
AST (U/L)	−0.15	0.16
ALT (U/L)	0.06	0.64
Alkaline phosphatase (U/L)	−0.12	0.35

*Note:* Statistically significant correlations are highlighted **in bold.**

Abbreviations: ALT, alanine aminotransferase; AST, aspartate aminotransferase.

On questionnaire (see Supplement) 52% (*n* = 41) of participants had comorbidities known to be made worse by increasing adiposity (27% [*n* = 21] asthma, 20% [*n* = 16] constipation, 25% [*n* = 16] obstructive sleep apnea, 20% [*n* = 16] diagnosed behavioral problems, 12% [*n* = 9] genitourinary problems, and 10% [*n* = 8] adenotonsillar hypertrophy). In addition, on initial physical examination, 45% (*n* = 36) of participants were found to have acanthosis nigricans, 30% (*n* = 24) to have lipomastia, and 5% (*n* = 4) to have a cervicodorsal hump.

### 3.3 Participant retention and referral

**FIGURE 4:**
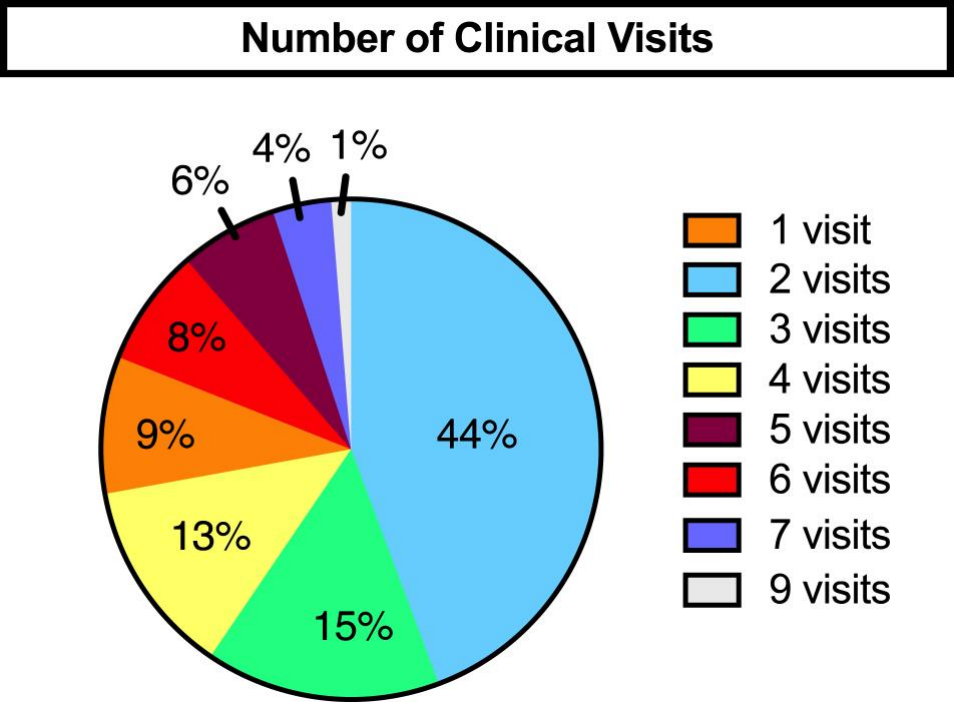
Number of visits completed by subjects. The initial visits with the physician and dietitian were classified as visit 1. Subsequent visits were defined as meeting with the primary care obesity MD and/or with the dietitian after the initial meeting. A return appointment for fasting blood tests obtained by a phlebotomist was not counted as a clinical visit and subjects who did not return for fasting blood work were considered as having had only one visit. All subjects had the opportunity to complete at least two visits and only 9% of subjects (orange segment) failed to do so. Those enrolled earlier had the opportunity to complete more visits. All subjects were enrolled for a sufficient period of time to generate at least two visits

As shown in Figure 4, over 90% of participants returned for at least two visits (defined as at least one visit with the primary FIT physician or registered dietitian after laboratory testing had been performed and discussed with the primary FIT physician) to the FIT program. The group discussed all patients. Other than three patients referred to ENT for evaluation of possible sleep apnea, no patient was referred to any subspecialty clinic outside of FIT for adiposity‐related comorbidities. Rates of retention for two visits or more (as opposed to at least four visits in FIT— one for the initial screen, one for fasting laboratory testing, and one to discuss laboratory results, and a subsequent visit) reported in other pediatric obesity programs^39^ ranged from approximately 50%–60% (*p* < 0.001 vs. FIT) for usual care^40^, ^41^ to 74% (*p* = 0.0016 vs. FIT) for enhanced care consisting of 2 h of in‐program lifestyle intervention per week for 12 weeks.^34^ As noted, there are, to our knowledge, no available data on referral rates of children with obesity to subspecialists within obesity programs and reports from primary care programs for referrals, besides to dietitians or pediatric obesity programs ranges from 7% (*p* < 0.05 vs. FIT) to 25% (*p* < 0.001 vs. FIT).^21^, ^36^

### 3.4 Research

Research efforts were productive with successful establishment of the database integrated with the electronic medical record, three nationally presented abstracts^42^, ^43^, ^44^ with young investigators as primary authors, two manuscripts published^45^, ^46^ with FIT investigators as senior authors, and one manuscript in preparation.^47^ FIT collaborated with two NIH R01 grants (DK097399 and DK107735 studying FTO SNP's and feeding behavior and pediatric nonalcoholic hepatic steatosis respectively), one NIH K award (DK110539, studying obesity genetics), a grant from Regeneron, Inc. looking for specific genotypes and phenotypes for a clinical trial, and an internal grant examining the precursors and definition of metabolic syndrome in children. Approximately 75% of families consented to participate in research via biobanking.

Means of establishing meaningful community outreach programs was also a research project within FIT. FIT investigators met with local community boards to develop a list of large primary care pediatric providers and other contacts within the community, and then with some of the large pediatric primary care group physicians to gather information as topics that they were interested in that were relevant to obesity in childhood. Finally, working with the Community Engagement Core Research (CECR) within the Columbia University Clinical Translational Science Award (CTSA) a community outreach program was designed to educate primary care pediatricians and family practitioners regarding co‐morbidity assessment of children who are overweight or obese (e.g., new hypertension, cholesterol, and vitamin D guidelines)^48^, ^49^, ^50^, ^51^, ^52^ and at what point subspecialty referral is warranted. The goal was to transfer some aspects of obesity care to the primary care provider while familiarizing them with resources and engaging them as collaborators in FIT. However, community‐based health provider attendance at these presentations was poor and further marketing development is clearly needed. Notably, FIT was unable to offer CME credits, which primary care physicians in the Columbia community indicated reported as a significant factor in considering whether or not to attend.

## 4 DISCUSSION

The primary FIT goal was to develop an effective clinical infrastructure minimizing barriers to patient participation in family‐based medical, nutritional, and exercise treatment as well as interdisciplinary research relevant to obesity in childhood. The primary outcome variables of this pilot study were participant retention and interdisciplinary research collaborations. The major findings of this pilot study were that providing a multispecialty program with a single primary obesity care provider was associated with a significantly higher patient retention rate and lower need for outside referrals while providing an excellent research venue and educational opportunity for early career fellows and faculty. The extremely high rate of adiposity‐related co‐morbidities even in young children with obesity are consistent with other studies and indicate that this study population is typical of other similarly‐aged groups of children with obesity.^53^, ^54^, ^55^

Other investigators have also noted higher short‐ and long‐term retention rates and/or other outcomes in interdisciplinary programs^56^, ^57^ supporting the idea the centralization of care to a single program or even single physician is beneficial. Burton et al.^55^ noted that children who remained in a 2‐year healthy lifestyle clinic showed a pronounced shift toward single provider, rather than multispecialty, follow‐up over time. Research collaboration between families and investigators was excellent and consent for biobanking and future contact was obtained from 75% of families and these collaborative efforts may also have improved retention.

FIT also established a research infrastructure and mineable database and integrated pediatric obesity treatment with translational research. A mineable de‐identified database was created which integrated questionnaire data with direct downloads from the hospital computer system. Growth curves, medical histories, and validated questionnaires regarding background genetic (family history), and behavioral (dietary and activity/leisure patterns) histories^52^ were obtained prior to initial visits. Ability to attract collaborators was excellent. FIT collaborated with two NIH R01 grants (DK097399 and DK107735 studying FTO SNP's and feeding behavior and pediatric nonalcoholic hepatic steatosis respectively), one NIH K award (DK110539, studying obesity genetics), a grant from Regeneron, Inc. looking for specific genotypes and phenotypes for a clinical trial, and an internal grant examining the precursors and definition of metabolic syndrome in children. As reported in Section 3, research productivity was high with three nationally presented abstracts^42^, ^43^, ^44^ with young investigators as primary authors, two manuscripts published^45^, ^46^ with FIT investigators as senior authors, and one manuscript in preparation.^47^ The involvement of the entire team in discussing the care of each participant may have precipitated research by creating greater familiarity for all team members with the entire population and with different research protocols.

The prevalence of adiposity‐related comorbidities in this population emphasizes the need for early intervention. Blood pressure was measured three times at each visit, but the prevalence of hypertension in this population was roughly twice what is commonly reported in children with obesity.^58^ The association of body fatness and/or waist circumference with insulin resistance,^59^, ^60^ dyslipidemia,^60^ hypovitaminosis D^61^ and NAFLD^62^ are well established, and the prevalence of these comorbidities in the study population are consistent with other studies.

FIT was a short‐term pilot program designed to assess rates of attrition of participants and collaborations between faculty in a novel setting that focused on a single primary care obesity physician with a multispecialty/interdisciplinary support team. The strengths of the program were that these assessment goals were met. The weaknesses were mainly due to the temporal and budgetary limitations of the program. The United States Preventive Services Taskforce (USPTF) recommendations^63^ were not met at multiple levels beyond those specified in Stage 1: Prevention Plus and Stage 2: Structured Weight Management. While a regular exercise family‐based program was established, fewer than 20% of participants actually attended and recommendations for 25 h of patient contact within 6 months were also not met. Numerous recommended resources including social workers, staff with training in motivational interviewing and in teaching of monitoring and reinforcement techniques and counselor for help with parenting skills, resolution of family conflict, or motivation were not available. Finally, it should also be emphasized that this was not a clinical trial and there was no assessment of intervention efficacy or program financial sustainability.

These issues should be addressable in longer‐term studies of this model hopefully utilizing innovations in telemedicine and other community outreach programming to meet the American Academy of Pediatrics guidelines for management of obesity in childhood.^64^ Spence et al.^65^ have suggested a standardized approach for the study of program attrition that would allow better comparison between methods. The adverse effects of obesity on clinical outcome in adults and children with COVID‐19^66^, ^67^ coupled with decreased access to clinical weight loss programs^68^, ^69^ further emphasize the need to further address obstacles related to programming for children with obesity.^70^

In summary, the FIT program demonstrated increased retention of patients in a pediatric obesity treatment program with fewer necessary outside referrals and was a successful venue in which to promote pediatric obesity research. Additional benefits were the academic interactions between multiple relevant specialists and the training of the next generation of physicians in the prevention and treatment of pediatric obesity. Fritner et al. reported that, in a survey of 1000 recent pediatric residency program graduates, 54% wanted more training in obesity prevention and treatment.^71^ The FIT model allows multispecialty training of house officers and junior faculty in multiple aspects of pediatric obesity research, prevention, and care, with emphasis on multi‐level treatment, without necessitating multispecialty clinic visits.

## CONFLICT OF INTERESTS

The authors declared no conflict of interest. The views expressed in this paper are those of the authors, and do not necessarily represent the positions of the NIH, the DHHS, or the Federal Government.
